# Sarcopenic Obesity Is Significantly Associated With Coronary Artery Calcification

**DOI:** 10.3389/fmed.2021.651961

**Published:** 2021-03-29

**Authors:** Goh Eun Chung, Hyo Eun Park, Heesun Lee, Min Joo Kim, Su-Yeon Choi, Jeong Yoon Yim, Ji Won Yoon

**Affiliations:** Internal Medicine, Seoul National University Hospital Healthcare System Gangnam Center, Seoul, South Korea

**Keywords:** sarcopenia, coronary artery calcification, obesity, atherosclerosis, cardiovascular risk

## Abstract

**Background:** An association between sarcopenic obesity and cardiovascular disease has been suggested. We investigated the relationship between sarcopenia and coronary atherosclerosis, taking into account the presence or absence of obesity in a health check-up population.

**Methods:** Data were reviewed for subjects who underwent bioelectrical impedance analysis (BIA) and coronary calcium scoring (CAC) computed tomography between January 2017 and December 2018. Appendicular skeletal muscle mass (ASM) was assessed using BIA. Sarcopenia was defined as reduction of muscle mass and calculated as ASM% (ASM/body weight) more than two standard deviations below the sex-specific mean for healthy young adults. CAC scores were dichotomized as low (<100) or high (≥100).

**Results:** Among 1,282 subjects (mean age, 58.1 years; 75.5% male), the prevalence of high CAC was 21%. When the study population was divided into four groups according to their obesity and sarcopenia status, the prevalence of high CAC in the sarcopenic-obesity (SO) group was significantly higher than in the other groups (40.7%, *P* < 0.001). After adjusting for age, sex, hypertension, diabetes, dyslipidemia, and creatinine, subjects with SO exhibited a significantly higher odds of a high CAC score, compared with the non-sarcopenic, non-obese group (odds ratio, 1.92; 95% confidence interval, 1.16–3.18, *P* = 0.011).

**Conclusion:** SO was significantly associated with CAC, independent of known risk factors for coronary artery disease. These findings suggest that sarcopenia and obesity may potentiate each other to increase atherosclerotic burden in coronary arteries, which may eventually lead to adverse cardiovascular events.

## Key Points

- Sarcopenic obesity is significantly associated with coronary artery calcification, independent of established risk factors for coronary artery disease.
- The combination of obesity and sarcopenia may be associated with an increased risk of coronary atherosclerosis, which may eventually lead to cardiovascular events.

## Introduction

Sarcopenia refers to an age-related decline in skeletal muscle mass and strength, with or without a reduction in physical performance (1). The importance of sarcopenia is based on its relationship with metabolic and cardiovascular diseases (2–4), as excess fat mass and reduced lean mass are associated with increased mortality. Thus, sarcopenia and obesity act interactively, resulting in various metabolic and functional impairments.

Sarcopenic obesity (SO) is the state in which sarcopenia and obesity coexist, presenting as both decreased muscle mass and increased adiposity. Previous studies have reported an association between SO and traditional cardiovascular risk factors, including metabolic syndrome, diabetes, and insulin resistance (5). However, the association between SO and cardiovascular outcomes remains unclear, as studies have shown conflicting results. Stephen and Janssen followed 3,366 community-dwelling elderly individuals with no baseline cardiovascular disease for 8 years. They reported that sarcopenia (defined as reduced muscle strength or mass) and obesity alone did not affect the risk of cardiovascular disease, but SO (defined by reduced muscle strength) was associated with a 23% increased risk of cardiovascular disease (6). In another study, Farmer et al. investigated the association of sarcopenia (assessed by grip strength) and obesity with cardiovascular disease and showed that obesity alone, sarcopenia alone, and SO were all associated with increased cardiovascular and all-cause mortality, as well as cardiovascular disease events (except sarcopenia alone) (7). These results indeed show that SO is associated with cardiovascular disease.

Coronary artery calcification (CAC) reflects the burden and severity of atherosclerosis in coronary arteries and is a well-known marker associated with adverse cardiovascular outcomes (8, 9). CAC is also used for individual risk stratification and to detect subclinical atherosclerosis. A population study reported a dose-dependent inverse relationship between lower relative muscle mass and CAC score (10). However, the clinical significance of sarcopenia, obesity, and SO in association with CAC remains to be elucidated. In this study, we aimed to investigate the relationship between sarcopenia and CAC, taking into account the presence or absence of obesity, in a health check-up population.

## Methods

### Study Population

This retrospective observational study included subjects who underwent routine health check-up at the Seoul National University Hospital Healthcare System Gangnam Center between January 2017 and December 2018. The subjects voluntarily attended for a general health check-up, while others were supported by their employer. They were mostly free of symptoms and voluntarily underwent examinations including bioelectrical analysis, CAC-scoring computed tomography (CT) and blood samplings on the same day. Of 1,304 potential study participants, 22 were excluded because of a previous myocardial infarction, past or current angina, or past or current congestive heart failure presumed secondary to coronary artery disease. Therefore, 1,282 subjects were included in the final analysis.

The study protocol followed the guidelines of the Declaration of Helsinki of 1975, as revised in 1983. The protocol was approved by the Institutional Review Board of Seoul National University Hospital (No. 1606-102-771). Informed consent was waived by the board since researchers accessed and analyzed only de-identified data.

### Measurement of Clinical and Laboratory Parameters

Data regarding past medical history, comorbidities, and medications were obtained using subject-recorded questionnaires. Blood pressure was measured twice, and mean values of the two measurements were reported. Hypertension was defined as a blood pressure ≥140/90 mmHg or receiving antihypertensive medications, and diabetes was defined as a fasting blood glucose ≥126 mg/dL or receiving glucose-lowering agents. Subjects taking lipid-lowering agents or with a total cholesterol ≥240 mg/dL were categorized as having hypercholesterolemia (11).

All blood samples were collected after a 12-h overnight fast. Laboratory tests included serum alanine aminotransferase, aspartate aminotransferase, total cholesterol, triglycerides, high-density lipoprotein cholesterol, fasting glucose, creatinine, and high-sensitivity C-reactive protein. All of these tests were performed using standard laboratory methods.

### Anthropometric Measurements

The methods employed in this study have been previously described in detail (12). Body weight and height were measured using a digital scale, and body mass index (BMI) was calculated by dividing weight (kg) by the squared value of height (m^2^). A well-trained person used a tape to measure the waist circumference at the midpoint between the lower costal margin and anterior superior iliac crest. For assessing body composition, bioelectrical impedance analysis (BIA) was performed using an InBody 720 Body Composition Analyzer (InBody Co., Ltd., Seoul, Korea). During this test, subjects remained in a standing position for 5–10 min with their legs slightly separated and their arms slightly abducted from the trunk. They were instructed to grasp the handles of the analyzer so each extremity contacted the electrodes. Multi-frequency measurements of impedance for each segment (including the trunk and four extremities) were obtained and used to estimate the appendicular skeletal muscle mass (ASM).

### Definitions of Sarcopenia and Obesity

ASM (kg) was calculated as the sum of the lean muscle mass in all four extremities. ASM% was calculated as ASM/weight (kg) ^*^ 100, as modified from Janssen et al. (13). Sarcopenia was defined as reduction of muscle mass and calculated as an ASM% more than two standard deviations (SDs) below the sex-specific mean for healthy young adults, according to nationwide health examinations of the Korean population (ASM% <29.0 in men and <22.9 in women) (14). Obesity was defined as a BMI ≥ 25 (kg/m^2^), according to the World Health Organization recommendation for the Asian-Pacific region (15). The subjects were classified into normal (control), obese, sarcopenic, and SO groups according to these definitions.

### Measurement of Coronary Artery Calcification

For all subjects, cardiac CT was performed for screening purposes at the patients' request. CT was conducted using a 256-slice multi-detector CT scanner (Brilliance iCT 256; Philips Medical Systems, Cleveland, OH, USA), with electrocardiogram-gated dose modulation. A standard scanning protocol was used, with 128 × 0.625 mm section collimation, 0.27 ms rotation time, 120 kV tube voltage, and 800 mA tube current. Data were reconstructed to generate 3-mm thick slices with a 400-ms acquisition window. The CAC score was calculated using a CT software program (Rapidia 2.8; INFINITT, Seoul, Korea) (16).

### Statistical Analysis

The outcome variable was CAC score, which was dichotomized as low (score <100) or high (≥100) (16). Continuous variables were expressed as mean ± SD, and categorical variables were expressed as number and percentage. Comparisons of continuous variables between groups were performed using Student's *t*-test or analysis of variance, and categorical variables were compared using chi-square test or Fisher's exact test. Logistic regression analysis was used to analyze the associations of sarcopenia, obesity, and SO with CAC score, while controlling for potential confounders. Multivariate logistic regression analysis was performed to determine the independent associations of sarcopenia, obesity, and SO with CAC, after adjusting for age, sex, hypertension, diabetes, dyslipidemia, and serum creatinine level. All statistical analyses were performed using SPSS 22.0 (SPSS Inc., Chicago, IL, USA), and *P* < 0.05 were considered statistically significant.

## Results

### Clinical Characteristics of Study Population

The mean age of our study population was 58.1 ± 9.3 years, and 75.5% of subjects were male. Among the 1,282 subjects, 21% had a high CAC score. Clinical characteristics according to CAC score are summarized in Table 1. Compared with the low CAC group, individuals with a high CAC score were older, were more frequently male, and had a higher BMI and higher prevalence of diabetes, hypertension, and dyslipidemia (*P* < 0.001). Total cholesterol and fasting blood glucose levels were also higher in individuals with a high CAC. The prevalence of sarcopenia was significantly higher in the high CAC group than in the low CAC group (16.5 vs. 7.4%, *P* < 0.05).

**Table 1:** Comparison of baseline characteristics according to coronary artery calcification score.

	**CACS <100 (*N* = 985)**	**CACS ≥100 (*N* = 297)**	***P*-value**
Age (years)	56.4 ± 8.8	62.5 ± 9.2	<0.001
Male, *n* (%)	699 (71.0)	265 (89.2)	<0.001
BMI (kg/m^2^)	24.4± 2.9	25.3 ± 3.2	<0.001
BMI ≥ 25 (kg/m^2^)	373 (37.9)	149 (50.2)	<0.001
Waist circumference (cm)	88.8 ± 8.3	91.7 ± 8.6	<0.001
ASM,% (kg)	30.7 ± 3.4	30.9 ± 3.1	0.418
Sarcopenia, *n* (%)	73 (7.4)	49 (16.5)	<0.001
**Comorbidities**			
Diabetes mellitus, *n* (%)	68/982 (6.9)	67 (22.6)	<0.001
Hypertension, *n* (%)	235/982 (23.9)	160 (53.9)	<0.001
Dyslipidemia, *n* (%)	209/983 (21.3)	133 (44.8)	<0.001
**Laboratory parameters**			
AST (IU/L)	28.2 ± 18.0	28.4 ± 16.0	0.893
ALT (IU/L)	26.8 ± 13.1	28.4 ± 15.9	0.093
Cholesterol (mg/dL)	195.0 ± 35.8	181.9 ± 44.4	<0.001
Triglyceride (mg/dL)	125.4 ± 74.6	120.7 ± 66.5	0.308
HDL-cholesterol (mg/dL)	53.1 ± 13.4	53.3 ± 14.6	0.816
Fasting glucose (mg/dL)	103.9 ± 17.7	113.0 ± 26.6	<0.001
Creatinine (mg/dL)	0.87 ± 0.2	0.91 ± 0.2	0.001
HS-CRP (mg/dL)	0.16 ± 0.6	0.15 ± 0.4	0.757

*Data are shown as the mean ± SD*.

*CACS, coronary artery calcification score; BMI, body mass index; AST, aspartate aminotransferase; ALT, alanine aminotransferase; ASM, appendicular muscle mass; HDL, high-density lipoprotein; HS-CRP, high sensitivity C-reactive protein*.

**Figure 1:**
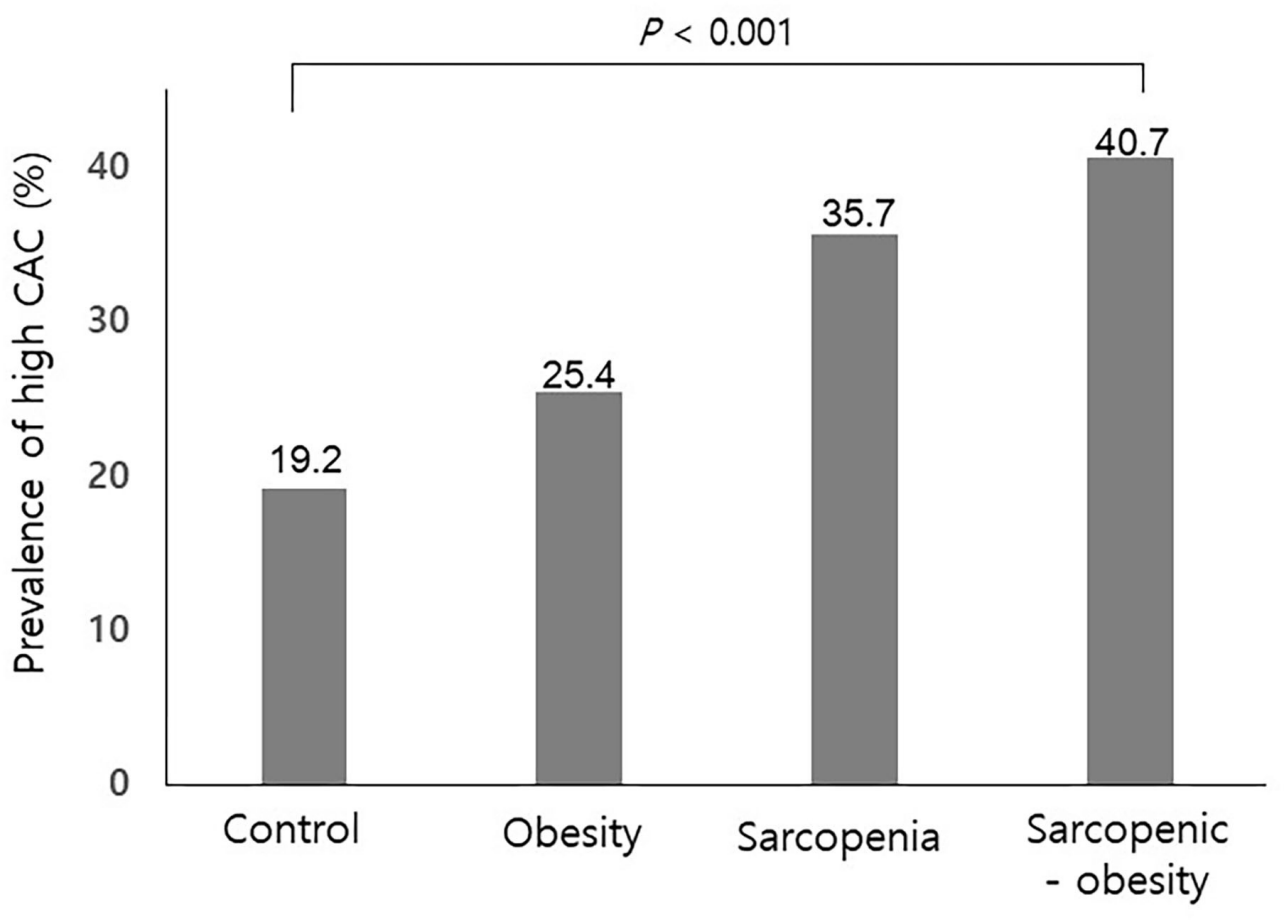
Prevalence of a high coronary artery calcification score according to sarcopenia with or without obesity. CAC, coronary artery calcification. *P* for trend.

Of the study subjects, 122 (9.5%) had sarcopenia, 522 (40.7%) were obese, and 108 (8.4%) had SO. Table 2 compares the four groups. Subjects with SO were more frequently male, had a higher prevalence hypertension, and exhibited higher serum AST, ALT, and triglyceride levels than those without SO. As shown in Figure 1, the prevalence of high CAC score was significantly higher in subjects with SO, compared with the other groups (*P* < 0.001 for trend).

**Table 2:** Comparison of baseline characteristics according to sarcopenia with/without obesity.

	**Control (*N* = 746)**	**Obesity (*N* = 414)**	**Sarcopenia (*N* = 14)**	**SO (*N* = 108)**	*P*-value^*^
Age (years)	58.6 ± 8.8	56.5 ± 9.9	64.5 ± 8.1	59.2 ± 10.2	<0.001
Male, *n* (%)	510 (68.4)	350 (84.5)	10 (71.4)	94 (87.0)	<0.001
Diabetes mellitus, *n* (%)	69/745 (9.3)	39/413 (9.4)	4 (28.6)	23/107 (21.5)	<0.001
Hypertension, *n* (%)	174/746 (23.3)	154/412 (37.4)	4 (28.6)	63/107 (58.9)	<0.001
Dyslipidemia, *n* (%)	187/745 (25.1)	122/414 (29.5)	6 (42.9)	27/107 (25.2)	0.205
AST (IU/L)	24.9 ± 14.2	31.8 ± 19.5	30.3 ± 12.4	36.1 ± 22.3	<0.001
ALT (IU/L)	26.2 ± 12.6	27.8 ± 13.2	26.7 ± 7.7	30.1 ± 14.7	0.013
Cholesterol (mg/dL)	191.8 ± 38.0	188.3 ± 38.6	175.3 ± 43.1	197.0 ± 44.6	0.055
Triglyceride (mg/dL)	106.2 ± 57.5	147.1 ± 79.4	135.5 ± 69.2	151.3 ± 96.7	<0.001
HDL cholesterol (mg/dL)	56.0 ± 13.9	49.4 ± 12.5	52.7 ± 21.7	49.7 ± 11.9	<0.001
Fasting glucose (mg/dL)	103.6 ± 17.7	107.6 ± 20.0	125.1 ± 38.0	116.7 ± 30.8	<0.001
Creatinine (mg/dL)	0.85 ± 0.2	0.92 ± 0.2	0.83 ± 0.1	0.88 ± 0.2	<0.001
HS-CRP (mg/dL)	0.14 ± 0.6	0.14 ± 0.2	0.07 ± 0.1	0.31 ± 0.8	0.041
CACS ≥100, *n* (%)	143 (19.2)	105 (25.4)	5 (35.7)	44 (40.7)	<0.001

*Data are shown as the mean ± SD*.

*SO, sarcopenia-obesity; HDL, high-density lipoprotein; HS-CRP, high sensitivity C-reactive protein; CACS, coronary artery calcification score*.

* *P-value for test of trend of odds*.

### Sarcopenia With or Without Obesity and Coronary Artery Calcium Score

An analysis of the relationship between sarcopenia (with or without obesity) and CAC score is shown in Table 3. Three different multivariate models were constructed, adjusting for traditional atherosclerosis risk factors. When adjusting for age and sex (Model I), obesity and SO were associated with an increased risk of coronary calcification [odds ratio (OR), 1.54; 95% confidence interval (CI), 1.13–2.11, and OR, 2.61; 95% CI, 1.64–4.16, respectively]. As shown in Models II and III, additional adjustments with other traditional cardiovascular risk factors attenuated the correlation between SO and CAC, but in all three models, SO remained a statistically and clinically significant parameter associated with increased odds of a high CAC score (Model II: OR, 1.92; 95% CI, 1.16–3.18; *P* = 0.009; Model III: OR, 1.92; 95% CI, 1.16–3.18; *P* = 0.011). However, the association with obesity alone no longer remained statistically significant after additional covariate adjustments (Model II: *P* = 0.058; Model III: *P* = 0.114; Table 3). When we performed stratified analysis according to sex and age groups, the association between SO and CAC was significant only in men (Table 4).

**Table 3:** Multivariate analyses of the risk for coronary artery calcification according to sarcopenia with or without obesity.

	**Model I**	***P-*value**	**Model II**	***P-*value**	**Model III**	***P-*value**
	**OR (95% CI)**		**OR (95% CI)**		**OR (95% CI)**	
Control	1 (ref)	<0.001^*^	1 (ref)	0.041^*^	1 (ref)	0.064^*^
Obesity	1.54 (1.13–2.11)	0.007	1.37 (0.99–1.90)	0.058	1.31 (0.94–1.82)	0.114
Sarcopenia	1.58 (0.47 ~ 5.29)	0.455	1.40 (0.36 ~ 5.43)	0.627	1.33 (0.36 ~ 4.97)	0.670
Sarcopenia with obesity	2.61 (1.64 ~ 4.16)	<0.001	1.92 (1.18–3.13)	0.009	1.92 (1.16–3.18)	0.011

*OR, odds ratio; CI, confidence interval*.

*Model I: adjusted for age and sex*.

*Model II: adjusted for age, sex, hypertension, and diabetes*.

*Model III: adjusted for age, sex, hypertension, diabetes, dyslipidemia, and creatinine*.

* *P-value for test of trend of odds*.

**Table 4:** Subgroup analysis of the risk for coronary artery calcification according to sarcopenia with or without obesity.

	**Men**		**Women**	
	**OR (95% CI)**	***P-*value**	**OR (95% CI)**	***P-*value**
Control	1 (ref)	0.035^*^	1 (ref)	0.443^*^
Obesity	1.31 (0.92–1.88)	0.138	1.49 (0.59–3.76)	0.400
Sarcopenia	0.99 (0.23–4.36)	0.993	3.57 (0.28–45.31)	0.326
Sarcopenia with obesity	2.20 (1.28–3.78)	<0.001	0.35 (0.04–3.08)	0.342
	**Age** **<** **65**		**Age** **≥** **65**	
Control	1 (ref)	0.463^*^	1 (ref)	0.479^*^
Obesity	1.22 (0.83–1.80)	0.312	1.06 (0.56–2.03)	0.855
Sarcopenia	1.18 (0.14–10.31)	0.879	1.87 (0.29–12.00)	0.509
Sarcopenia with obesity	1.58 (0.10–0.43)	0.134	1.96 (0.80–4.78)	0.142

*OR, odds ratio; CI, confidence interval*.

*Adjusted for age, sex, hypertension, diabetes, dyslipidemia, and creatinine*.

* *P-value for test of trend of odds*.

We then evaluated the role of obesity in the association between CAC and sarcopenia. The prevalence of obesity was 40.7% in our study population. In multivariate analysis, obesity alone was significantly associated with an increased risk of CAC (Supplementary Table 1). Thus, we divided obese subjects into obesity without sarcopenia and obesity with sarcopenia (i.e., SO). In the age- and sex-adjusted Model I, obesity without sarcopenia and SO were both associated with an increased odds of a high CAC score (OR, 1.53; 95% CI, 1.12–2.09 and OR, 2.58; 95% CI, 1.62–4.11, respectively). However, when adjusted for additional cardiovascular risk factors, only SO associations remained statistically significant (Model II: OR, 1.91; 95% CI, 1.17–3.13; *P* = 0.010 and Model III: OR, 1.91; 95% CI, 1.15–3.16; *P* = 0.012; Table 5).

**Table 5:** Multivariate analyses of the risk for coronary artery calcification according to obesity with or without sarcopenia.

	**Model I**	***P-*value**	**Model II**	***P-*value**	**Model III**	***P-*value**
	**OR (95% CI)**		**OR (95% CI)**		**OR (95% CI)**	
Control	1 (ref)	<0.001^*^	1 (ref)	0.018^*^	1 (ref)	0.029^*^
Obesity without sarcopenia	1.53 (1.12–2.09)	0.008	1.36 (0.98–1.88)	0.062	1.30 (0.93–1.81)	0.121
Obesity with sarcopenia	2.58 (1.62 ~ 4.11)	<0.001	1.91 (1.17–3.13)	0.010	1.91 (1.15–3.16)	0.012

*CAC, coronary artery calcification score; OR, odds ratio; CI, confidence interval*.

*Model I: adjusted for age and sex*.

*Model II: adjusted for age, sex, hypertension, and diabetes*.

*Model III: adjusted for age, sex, hypertension, diabetes, dyslipidemia, and creatinine*.

* *P-value for test of trend of odds*.

## Discussion

As shown in the present study, patients with SO have a higher risk of CAC. A significant association was found between SO and a high CAC score, independent of established risk factors for coronary artery disease. These findings suggest that the combination of obesity and sarcopenia may be associated with an increased risk of developing coronary atherosclerosis.

Reported prevalence of sarcopenia has varied according to the definitions used and populations studied. The prevalence has ranged from 5 to 13% in patients above 60 years of age (17, 18). Results of previous studies of Korean populations have reported sarcopenia prevalence of 5.1% in men and 14.2% in women aged ≥60 years (19), and 9.7% in men and 11.8% in women aged ≥65 years (14). These values are generally similar to the results of the current study, in which we found a 9.7% overall prevalence of sarcopenia, using the definition modified from Janssen et al. (13), in our Korean subjects with no known coronary artery disease and a mean age of 58 years. The prevalence of SO was 8.4% in our study population, which was similar to the prevalence reported in one previous study (5.1% in men and 12.5% in women aged ≥60 years) (19) and lower than the values reported in another study (18.3 and 26.6% in women) (20). Differences in prevalence likely reflect varying definitions of SO and different study populations.

Obesity is a well-established major risk factor for cardiovascular disease (21–23). However, obesity defined by BMI does not account for wide variations in body fat distribution and may not correspond to the same degree of fatness or associated health risks in different individuals and populations. A previous study reported that sarcopenia but not excess weight or total caloric intake was associated with subclinical atherosclerosis in elderly patients (24). Thus, considering sarcopenia in addition to obesity may increase the accuracy of predicting cardiovascular disease risk.

Various studies have investigated the clinical significance of sarcopenia and SO in relation to cardiovascular disease (5). In a Japanese population study, sarcopenia was associated with greater arterial stiffness in women, suggesting that sarcopenia is associated with risk factors for atherosclerosis (25). Health interview survey population data likewise showed that sarcopenia was independently associated with cardiovascular events (3). Moreover, SO was associated with a higher risk for dyslipidemia (20), metabolic syndrome (26), diabetes (27), and hypertension (28) than sarcopenia or obesity alone. In our study, non-sarcopenic obese and non-obese sarcopenic groups did not have a significantly increased odds of a high CAC score in multivariate analysis. This suggests that obesity and sarcopenia alone may be insufficient to significantly increase the risk of developing atherosclerosis. Although not statistically significant, the non-sarcopenic obese and non-obese sarcopenic groups showed similar increases in OR for a high CAC score, and the SO group exhibited an even higher OR, suggesting that obesity and sarcopenia may exert additive or even synergistic effects on the development of CAC. In this study, the association between SO and CAC was significant only in men, suggesting the different effect depending on gender.

CT-based screening evaluation has become an important role in detecting subclinical atherosclerosis, which has shown incremental predictive value over known risk factors. CAC is a useful tool for individualized-risk stratification and outcome prediction (29–31). The novel finding of the current study is the independent association between SO and CAC, which was not seen with sarcopenia alone or obesity alone. SO itself was significantly associated with CAC, reflecting greater risk and burden of atherosclerotic changes in people with SO.

The underlying mechanism of the close link between SO and coronary atherosclerosis is not fully understood. Loss of skeletal muscle reduces the mass of the primary tissue responsible for insulin-mediated glucose disposal (17, 32) and promotes insulin resistance, which plays a key role in the pathogenesis of atherosclerosis. Increased muscle strength has been associated with reduced blood pressure and improved hemodynamics (33), suggesting that muscle exerts a protective role in the development of atherosclerosis. In addition, obesity promotes inflammation by increasing pro-inflammatory cytokines, such as tumor necrosis factor-alpha and interleukin-6, and increases cardiovascular disease risk (34, 35). These inflammatory cytokines induce muscle atrophy and are found quite consistently in sarcopenia (34, 35). In a previous study, cellular interleukin-6 production and serum insulin-like growth factor-1 levels were significant predictors of sarcopenia, suggesting that they play an important role as inflammatory cytokines (36). Thus, obesity may aggravate sarcopenia, and sarcopenia may exacerbate obesity by decreased metabolic rate and myokine deficiency (37); this vicious cycle may exert synergistic detrimental effects on cardiovascular risk.

## Limitations

This study has some limitations. First, its cross-sectional design limits the ability to verify causality. Thus, we could not infer causal relationships from this study. Second, BIA is not the gold standard method to evaluate muscle mass. However, it is a very useful and practical tool for screening purposes to evaluate muscle and fat mass, especially in routine clinical evaluations. Third, although sarcopenia is not simply a measure of skeletal mass but also of strength which is a component of the new diagnostic criteria for sarcopenia (38), we could not evaluate muscle strength (e.g., grip strength) or physical performance in this study. Lastly, our study population, who underwent health evaluations upon their own initiative, may not represent the general Korean population. Our results, therefore, should be interpreted with caution.

## Conclusion

Subjects with SO have a higher risk for coronary atherosclerosis, as exemplified by higher CAC scores. Obesity alone and sarcopenia alone were not associated with high CAC scores. These results suggest that sarcopenia and obesity may potentiate each other to increase atherosclerotic burden in coronary arteries, which may eventually lead to adverse cardiovascular events. Further studies are warranted to understand the mechanism of association between SO and atherosclerosis and verify a causal relationship between SO and coronary artery calcification and disease.

## Data Availability Statement

The raw data supporting the conclusions of this article will be made available by the authors, without undue reservation.

## Ethics Statement

The studies involving human participants were reviewed and approved by Institutional Review Board of Seoul National University Hospital. Written informed consent for participation was not required for this study in accordance with the national legislation and the institutional requirements.

## Author Contributions

GC, HP and JWY conceived the idea, determined the study design, collected the data, and drafted and revised the manuscript. HL and MK collected the data, performed the statistical analysis, and revised the manuscript. S-YC and JYY collected and reviewed the data and revised the manuscript. All authors contributed to the article and approved the submitted version.

## Conflict of Interest

The authors declare that the research was conducted in the absence of any commercial or financial relationships that could be construed as a potential conflict of interest.
