# Supplementary material for: Sarcopenic Obesity Is Significantly Associated With Coronary Artery Calcification
Source: Front Med (Lausanne). 2021 Mar 29;8:651961. doi: 10.3389/fmed.2021.651961 (PMC8039284; doi:10.3389/fmed.2021.651961)
Supplement: Supplementary file 1 [file Table_1.DOC]

**Supplementary Table 1.** Univariate analyses of the risk for coronary artery calcification

| Variables | Univariate OR  (95% CI) | *P-*value | Multivariate OR  (95% CI) | *P-*value |
| --- | --- | --- | --- | --- |
| Age (years) | 1.08 (1.06-1.10) | *<0.001* | 1.08 (1.06-1.10) | *<0.001* |
| Female | 0.28 (0.19-0.42) | *<0.001* | 0.79 (0.11-0.32) | *<0.001* |
| Body mass index (kg/m^2^) | 1.11 (1.06-1.15) | <0.001 |  |  |
| BMI ≥ 25 (kg/m^2^) | 1.65 (1.27-2.15) | <0.001 | 1.42 (1.04-1.93) | <0.026 |
| Waist circumference (cm) | 1.04 (1.03-1.06) | *<0.001* |  |  |
| Diabetes mellitus | 3.25 (2.23-4.73) | *<0.001* | 1.93 (1.26-2.97) | *0.003* |
| Hypertension | 3.51 (2.75-4.49) | *<0.001* | 2.13 (1.56-2.93) | *<0.001* |
| Dyslipidemia | 2.77 (2.15-3.57) | <0.001 | 1.66 (1.19-2.31) | 0.003 |
| AST (IU/L) | 1.00 (1.00-1.01) | 0.893 |  |  |
| ALT (IU/L) | 1.01 (1.00-1.02) | 0.103 |  |  |
| Cholesterol (mg/dL) | 0.99 (0.99-0.99) | <0.001 |  |  |
| Triglycerides (mg/dL) | 1.00 (1.00-1.00) | 0.338 |  |  |
| HDL cholesterol (mg/dL) | 1.00 (0.99-1.01) | 0.807 |  |  |
| Fasting glucose (mg/dL) | 1.02 (1.01-1.03) | <0.001 |  |  |
| Creatinine (mg/dL) | 3.71 (1.62-8.53) | 0.002 | 0.81 (0.24-2.75) | 0.740 |
| Hs-CRP (mg/dL) | 0.96 (0.72-1.30) | 0.808 |  |  |

OR, odds ratio; CI, confidence interval; BMI, body mass index; AST, aspartate aminotransferase; ALT, alanine aminotransferase; HDL, high-density lipoprotein; HS-CRP, high sensitivity C-reactive protein.

Adjusted for age, sex, obesity, diabetes, hypertension, dyslipidemia and creatinine
